# High Mobility Thin Film Transistors Based on Amorphous Indium Zinc Tin Oxide

**DOI:** 10.3390/ma10070702

**Published:** 2017-06-26

**Authors:** Imas Noviyana, Annisa Dwi Lestari, Maryane Putri, Mi-Sook Won, Jong-Seong Bae, Young-Woo Heo, Hee Young Lee

**Affiliations:** 1 School of Materials Science and Engineering, Yeungnam University, Gyeongsan 38541, Korea; noviyanaimas@gmail.com (I.N.); annisadeel@gmail.com (A.D.L.); maryane.putri@gmail.com (M.P.); 2 Busan Center, Korea Basic Science Institute, Busan 46742, Korea; mswon@kbsi.re.kr (M.-S.W.); jsbae@kbsi.re.kr (J.-S.B.); 3 School of Materials Science and Engineering, Kyungpook National University, Daegu 41566, Korea; ywheo@knu.ac.kr

**Keywords:** amorphous oxide, thin film transistor, indium zinc tin oxide, RF magnetron sputtering, high field effect mobility, 73.61.Jc, 73.61.-r, 81.05.Gc

## Abstract

Top-contact bottom-gate thin film transistors (TFTs) with zinc-rich indium zinc tin oxide (IZTO) active layer were prepared at room temperature by radio frequency magnetron sputtering. Sintered ceramic target was prepared and used for deposition from oxide powder mixture having the molar ratio of In_2_O_3_:ZnO:SnO_2_ = 2:5:1. Annealing treatment was carried out for as-deposited films at various temperatures to investigate its effect on TFT performances. It was found that annealing treatment at 350 °C for 30 min in air atmosphere yielded the best result, with the high field effect mobility value of 34 cm^2^/Vs and the minimum subthreshold swing value of 0.12 V/dec. All IZTO thin films were amorphous, even after annealing treatment of up to 350 °C.

## 1. Introduction

Transparent amorphous oxide semiconductors have received much attention for such applications as thin film transistor (TFT) devices in liquid crystal displays (LCD), organic light emitting diodes (OLED), and transparent displays. In 2004, Hosono et al. made a breakthrough in replacing hydrogenated amorphous silicon (a-Si:H) and low-temperature polysilicon (LTPS) devices with amorphous oxide semiconductor in the fabrication of thin film transistors which were widely used in various display panels [1]. More recently, a-Si:H semiconductor has been excluded by most manufacturers because of its poor mobility, degradation under electrical bias stress, and instability under illumination [2,3,4,5]. In particular, LTPS is mostly used in active-matrix organic light emitting diode (AMOLED) displays with field effect mobility value of up to 100 cm^2^/Vs. However, despite its high mobility characteristics, LTPS usually showed relatively large threshold voltage variation [6,7,8]. In contrast, TFTs based on metal oxide channel layer created a whole new area to explore with such advantages as simpler manufacturing process with good characteristics including high on-current and low off-current [1,9,10].

A lot of amorphous semiconductors have been studied for possible TFT applications, e.g., zinc oxide (ZnO) [11], indium zinc oxide (IZO) [12], zinc tin oxide (ZTO) [13] and indium gallium zinc oxide (IGZO) [1]. Since Arai reported the amorphous indium zinc tin oxide (a-IZTO) with good field effect mobility in the range of ~30 cm^2^/Vs, it has attracted some attention to see if there exists the possibility of an alternative to a-IGZO [6]. IZTO is a ternary oxide semiconductor which is known to exhibit good electrical conductivity, high transparency, and high mobility, making it a promising candidate for further enhancement of the performance of display technologies [6]. In the IZTO system, both indium and tin have similar electron configurations with about the same conduction bands, which then allows electrons to move easier and faster, even in amorphous state [14].

In this study, the electrical and optical properties of IZTO thin films were examined for the films deposited from a ceramic target with the nominal chemical composition corresponding to 40 at % indium, 50 at % zinc, and 10 at % tin on the metallic component basis. The deposition of IZTO thin films was conducted using radio frequency (RF) magnetron sputtering as reported earlier by our group [15,16,17]. The variation of the electrical properties and TFT performance with annealing treatment was investigated in detail.

## 2. Experimental Section

Zinc-rich IZTO ceramic target with the metal ratio of In:Zn:Sn = 40:50:10 at % was prepared using the conventional mixed-oxide process. IZTO thin films were then sputter-deposited onto 15 mm × 15 mm-square commercial glass in order to observe the transparency and morphology of the films. Top-contact bottom-gate TFTs were fabricated where an IZTO active layer was deposited onto n^++^ heavily-doped silicon wafer with 200 nm-thick SiO_2_ gate insulating layer. Deposition of IZTO films was conducted using RF magnetron sputtering at room temperature with RF power of 125 W and working pressure of 5 × 10^−3^ Torr. Prior to deposition, the vacuum chamber was evacuated to a base pressure of 2 × 10^−5^ Torr or below. The deposition time was kept for 3 min to obtain the channel layer thickness of around 50 nm. During deposition, oxygen acted as ambient gas where O_2_:Ar ratio was 5%:95% while the gas flow rate was fixed at 20 sccm. After deposition, the films were annealed at temperature in the range of 150–350 °C for 30 min in air inside of the tube furnace. Titanium and copper bilayer metallic films were subsequently deposited as source and drain contacts using an e-beam evaporator through shadow mask with width and length dimensions of 350 μm and 150 μm, respectively. The structural and surface topography were characterized and confirmed by X-ray diffraction (XRD, Rigaku D-500) and atomic force microscopy (AFM, Nanoscope IIIA). The X-ray photoelectron spectroscopy (XPS) study was performed using an XPS system (Thermo Fisher Scientific K-Alpha, Waltham, MA, USA) with monochromated Al K*α* X-ray source (*hν* = 1486.6 eV) at a spot size of 400 μm in diameter with charge compensation. Survey spectra were obtained at pass energy of 200 eV and a resolution of 1 eV, and high-resolution spectra were acquired at pass energy of 30 eV and a resolution of 0.1 eV. All of the obtained binding energies (BEs) were compensated with that of adventitious carbon (C 1s) core level peak at 284.6 eV as a reference [18]. The Avantage software provided by the manufacturer was used for controlling the spectrometer, analyzing the spectra, and the deconvolution of O 1s core level spectra.

The electrical properties of the IZTO films and TFTs were characterized using Hall effect measurement (Ecopia HMS-5000, Anyang, Republic of Korea) and I–V measurement (Keithley 4200-SCS, Beaverton, OR, USA). The optical transmittance of the films across visible spectrum was observed using ultraviolet-visible spectrophotometer (UV/Vis/NIR spectrophotometer, Cary 5000, Agilent, Santa Clara, CA, USA).

## 3. Results and Discussion

**Figure 1:**
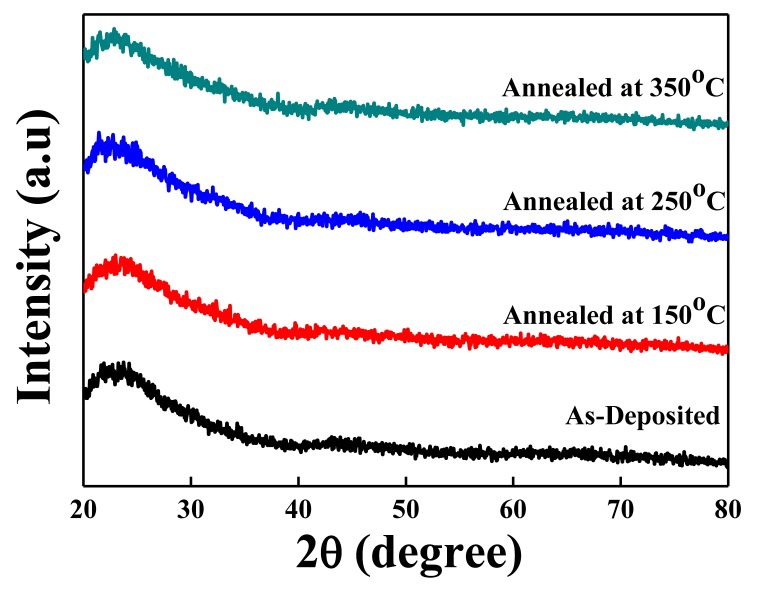
X-ray diffraction patterns of the indium zinc tin oxide (IZTO) films deposited at room temperature onto glass substrates at various annealing temperatures in the range of 150–350 °C.

X-ray diffraction patterns of the IZTO films deposited onto glass substrates at room temperature by RF magnetron sputtering are shown in Figure 1. The amorphous nature is clearly seen in all samples. This commonly happens in many multicomponent complex mixed oxide films where the crystallization energy is considerably higher than the thermal energy available at room temperature. Similar results were reported elsewhere [19,20], where IZTO films with low zinc content deposited at room temperature remained in amorphous state [20]. The crystallinity of the films is known to be affected by the processing variables, such as gas ambient, deposition temperature, working pressure, annealing temperature, and chemical composition [17,18,19]. However, even after annealing with temperature of up to 350 °C, no particular diffraction peaks corresponding to crystalline phases were observed from all IZTO films we had prepared. Furthermore, the surface topography observed using AFM revealed that subsequently, all films showed very smooth and uniform surface, which is very important for TFT application to minimize defects at the interlayers [21]. There was no prominent change in root-mean-square surface roughness values (R_q_), which increased from about 0.2 nm to 0.3 nm for all IZTO films as the annealing temperature increased, as summarized in Table 1.

**Figure 2:**
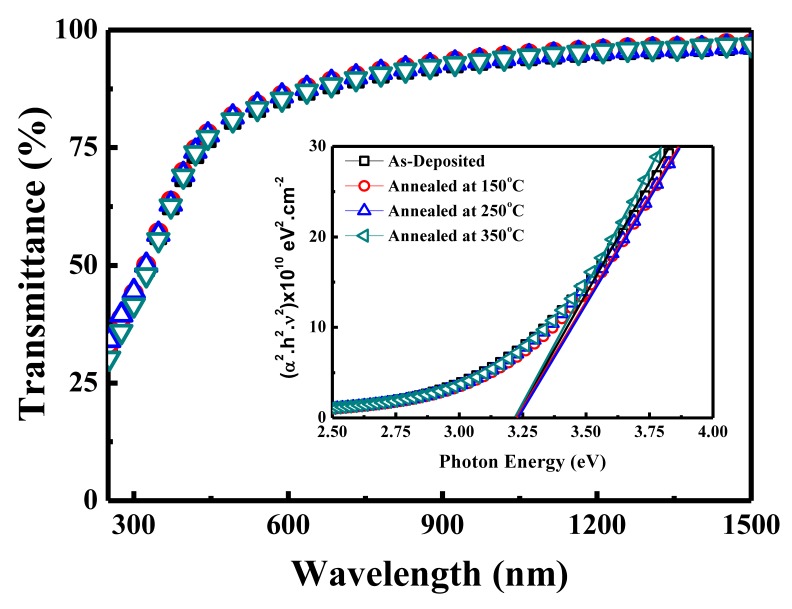
Optical properties of IZTO films deposited at room temperature onto glass substrates at various annealing temperatures in the range of 150–350 °C.

The optical transmittance was determined by taking the average value in the visible light region ranging between 400 nm and 700 nm in wavelength. Figure 2 illustrates the optical transmittance of the IZTO films deposited on glass substrate. Among all films, as-deposited IZTO film showed the lowest average transmittance of about 84%. The band gap energy value of IZTO films was estimated from the inset in Figure 2, which was done by extrapolating the linear part of *hν* versus (*αhν*)^2^ graph to the *x* axis according to Tauc equation [22,23]. The average optical band gap energy value was estimated at 3.25 eV, while there was no significant difference, even after annealing treatment. An optical band gap value of about 3 eV was reported from IZTO with composition of 50 at % zinc and 30 at % indium [20], as increasing indium and zinc deteriorated the optical transmittance and decreased the optical band gap energy of IZTO thin films [15,20]. Nevertheless, a shift in band gap energy and average transmittance values was reported elsewhere by composition variation of IZTO [15]. This is why an annealing treatment did not alter the band gap value of the IZTO film. Overall, the optical properties of all IZTO films exhibited high average transmittance above 80% and relatively high band gap energy, which is desirable for transparent display application.

Table 2 summarizes the typical electrical property data (i.e., carrier concentration and resistivity) of IZTO films. The carrier concentration value increased, while resistivity value decreased with the increase of annealing temperature. Lower values of resistivity led to an active layer with more electrons, resulting in the threshold voltage shift to the negative direction [24].

**Figure 3:**
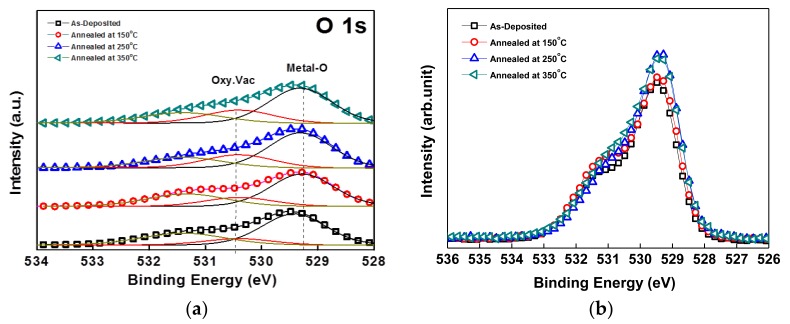
X-ray photoelectron spectroscopy (XPS) spectra of (**a**) detailed and (**b**) O 1s for IZTO films at various annealing temperatures in the range of 150–350°C. Metal-O: metal oxide; Oxy. Vac: oxygen vacancy.

Table 3 shows relative peak area ratio and binding energy of the IZTO thin films deposited by RF magnetron sputtering on silicon substrates at various annealing temperatures in the range of 150–350 °C. The related binding energies existed in metal-oxide (In-O, Zn-O, and Sn-O), oxygen vacancy (Oxy. Vac), and impurities such as hydroxides (O-OH) known as trapping sites on the interface of the TFT [25]. Based on the detailed O 1s XPS spectra of IZTO films shown in Figure 3a, the oxygen vacancy peak existed at the binding energy of 530.4 eV. It was seen that the oxygen vacancy tended to increase slightly with the increase of annealing temperature. This rather unusual result could only be explained by the rearrangement of oxygen ions in the film to thermodynamically more stable positions, thereby yielding slightly more oxygen vacancies. However, as shown in Table 2, the increase of electron concentration is much higher than the increase of oxygen vacancy, and thereby is not just because of the increase of oxygen vacancy concentration but rather because of the increase of singly- or doubly-ionized oxygen vacancy concentration yielding mobile electrons. As-deposited films should have a greater number of neutral oxygen vacancies with the two electrons trapped at or near the vacancy and does not contribute to mobile carriers [26]. In turn, this electron concentration increase brought the negative shift of the threshold voltage to be further explained below.

**Figure 4:**
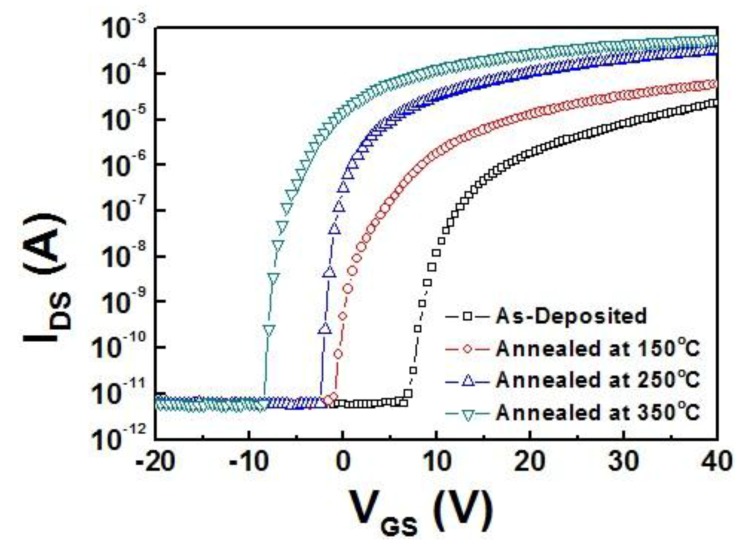
Transfer curve of IZTO thin film transistors deposited at various annealing temperatures in the range of 150–350 °C.

To investigate TFT device performance based on IZTO semiconductor channel layer, the transistor prepared using heavily-doped silicon wafer substrate was examined in n-channel mode. Figure 4 depicts the transfer characteristic of IZTO TFT device deposited by RF magnetron sputtering with annealing temperature variation. It is seen that field effect mobility (μ_FE_), on/off current ratio (I_on/off_), and subthreshold swing (SS) values improved as annealing temperature increased from 150 °C to 350 °C, as shown in Table 4. Threshold voltage (V_T_) value tended to shift to zero voltage by controlling the carrier concentration of IZTO channel layer with the increasing annealing temperature. As the carrier concentration increased, the threshold voltage and subthreshold swing values shifted to more desirable values [27,28]. The enhancement of TFT performance was noticed by lowering resistivity and reducing SS values of IZTO channel layer with increasing annealing temperature, which might be mainly due to oxygen diffusion from IZTO layer and rearrangement of molecular bonding during annealing process, thus inducing the acceleration of electrons to pass through channel region between source and drain [29].

Our TFT devices made from a zinc-rich IZTO channel layer demonstrated excellent performance with a field effect mobility value of 34 cm^2^/Vs, which is higher than the values reported by other research groups [24,30]. It is also noticed that interface defect concentration (N_T_) value, which was estimated from SS value, was reduced with increasing annealing temperature. It is obvious the mobility of charge carriers would then be improved as electrons travel from source to drain through the a-IZTO channel layer [19].

**Figure 5:**
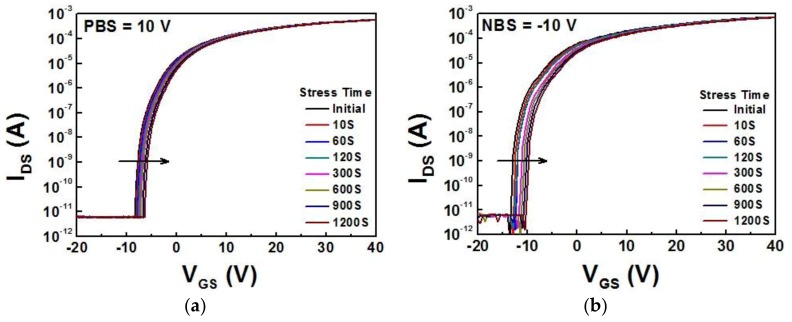
Transfer curves of amorphous IZTO (a-IZTO) thin film transistors annealed at 350 °C as a function of stress time under (**a**) positive bias stress (PBS) and (**b**) negative bias stress (NBS).

The stability of the a-IZTO thin film transistor annealed at 350 °C was explored under both positive bias stress (PBS) and negative bias stress (NBS). The tests were performed at drain voltage (V_DS_) of ±10 V with stress time of up to 1200 s. As shown in Figure 5, the transfer characteristics curve shifted to the positive direction, and thus threshold voltage also changed to the positive direction with the increase of bias stress time. This voltage shift of transfer characteristics was attributed to electron trapping at the gate/insulator interface in n-type TFTs [31]. The threshold voltage shift under PBS is about +1.9 V while the NBS is about +3.1 V. Higher bias instability of a-IZTO thin film transistor would be expected to decrease further by applying passivation layer to prevent humidity [32].

## 4. Summary and Conclusions

Room-temperature-deposited IZTO films remained uniform amorphous phase even after annealing at temperatures of up to 350 °C, indicating that the enhancement of TFT performance was not due to the crystallization of the IZTO layer. IZTO films deposited by RF magnetron sputtering showed transparency values higher than 84%, regardless of the annealing treatment across the visible light range, which is desirable for transparent electronic device applications. It was found that annealing treatment affected TFT parameters in such a way as to increase the carrier mobility and on/off current ratio, and to decrease the sub-threshold swing value. The threshold voltage value also shifted to the negative direction, and the carrier concentration value increased upon annealing. Interface defect concentration also reduced, resulting in the movement of more electrons without being trapped between the active layer and the source or drain electrode. However, stability improvement under bias stress still remains as an issue to enhance the performance of a-IZTO thin film transistors in the near future.

## Figures and Tables

**Table 1:** Root-mean-square values of the IZTO films from atomic force microscopy (AFM) data.

Annealing Temperature	R_q_ (nm)
As-Deposited	0.21
150 °C	0.31
250 °C	0.23
350 °C	0.33

**Table 2:** Electrical property data of the IZTO thin films deposited by RF magnetron sputtering on glass substrates at various annealing temperatures in the range of 150–350 °C.

Annealing Temperature	Carrier Concentration (cm^−3^)	Resistivity (Ω·cm)
As-Deposited	5.4 × 10^16^	10
150 °C	6.7 × 10^16^	9.5
250 °C	7.2 × 10^17^	0.60
350 °C	1.0 × 10^18^	0.33

**Table 3:** Relative peak area ratio and binding energy of the IZTO thin films deposited by RF magnetron sputtering on silicon substrates at various annealing temperatures in the range of 150–350 °C.

Annealing Temperature	Binding Energy (eV)			Peak Area (%)	
	Metal-Oxide	O-OH	Oxy. Vac	Metal-Oxide	Oxy. Vac
As-Deposited	529.42	531.39	530.40	82	18
150 °C	529.28	531.33	530.40	77	23
250 °C	529.32	531.34	530.40	75.8	24.2
350 °C	529.31	531.39	530.40	71.2	28.8

**Table 4:** Summary of transistor parameters of IZTO thin film transistors (TFTs) deposited by RF magnetron sputtering at various annealing temperatures in the range of 150–350 °C.

Annealing Temperature	µ_FE_ (cm^2^/Vs)	I_on/off_ (A)	V_T_ (V)	SS (V/dec)	N_T_ (cm^−2^)
As-Deposited	2.5	~10^6^	18.6	1.22	2.1 × 10^12^
150 °C	4.0	~10^7^	4.6	0.38	5.8 × 10^11^
250 °C	14	~10^7^	−0.8	0.15	1.6 × 10^11^
350 °C	34	~10^8^	−4.6	0.12	1.1 × 10^11^
